# Supplementary material for: Origin of Ferroelectricity in BiFeO3-Based Solid Solutions
Source: Nanomaterials (Basel). 2022 Nov 24;12(23):4163. doi: 10.3390/nano12234163 (PMC9740055; doi:10.3390/nano12234163)
Supplement: Supplementary file 1 [file nanomaterials-12-04163-s001.zip › Supplementary_Figures.pdf]

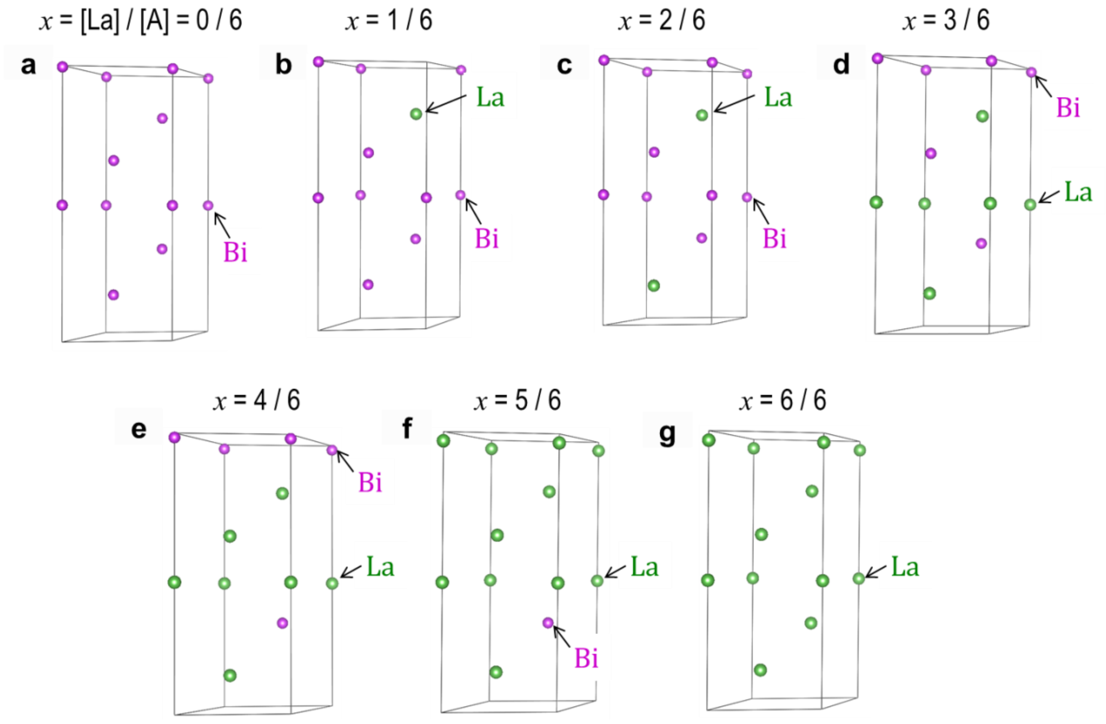

Figure S1. Bi and La arrangement on the A site in the rhombohedral system with $Bi_{1-x}La_xFeO_3$.

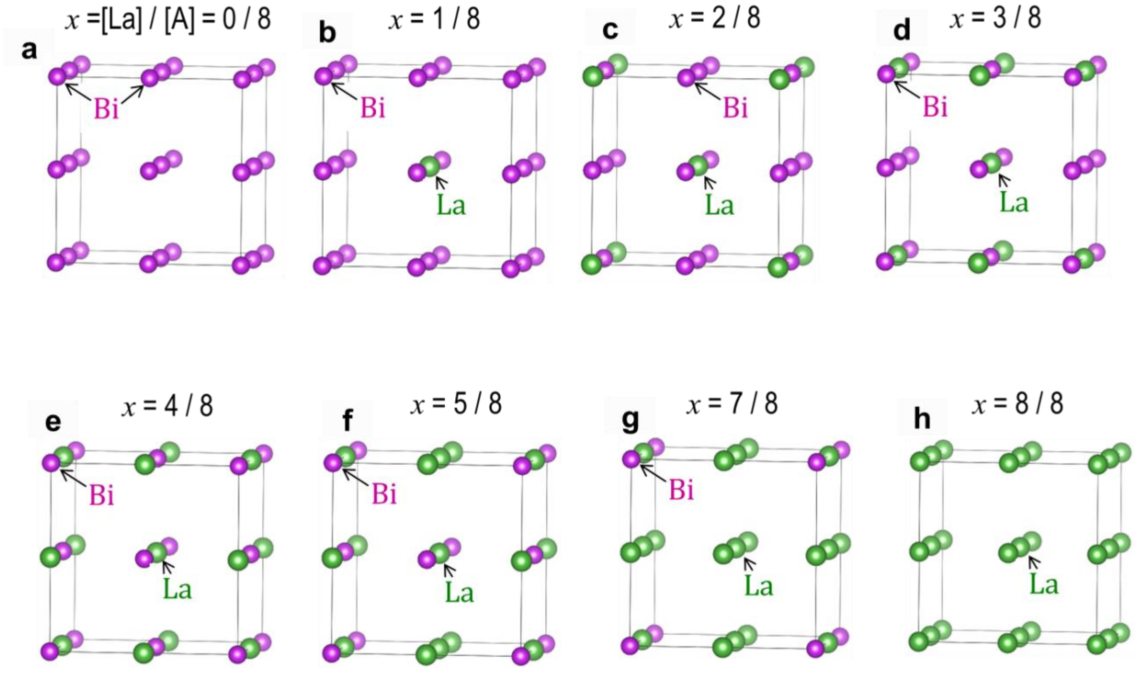

Figure S2. Bi and La arrangement on the A site in the tetragonal system with $Bi_{1-x}La_xFeO_3$.

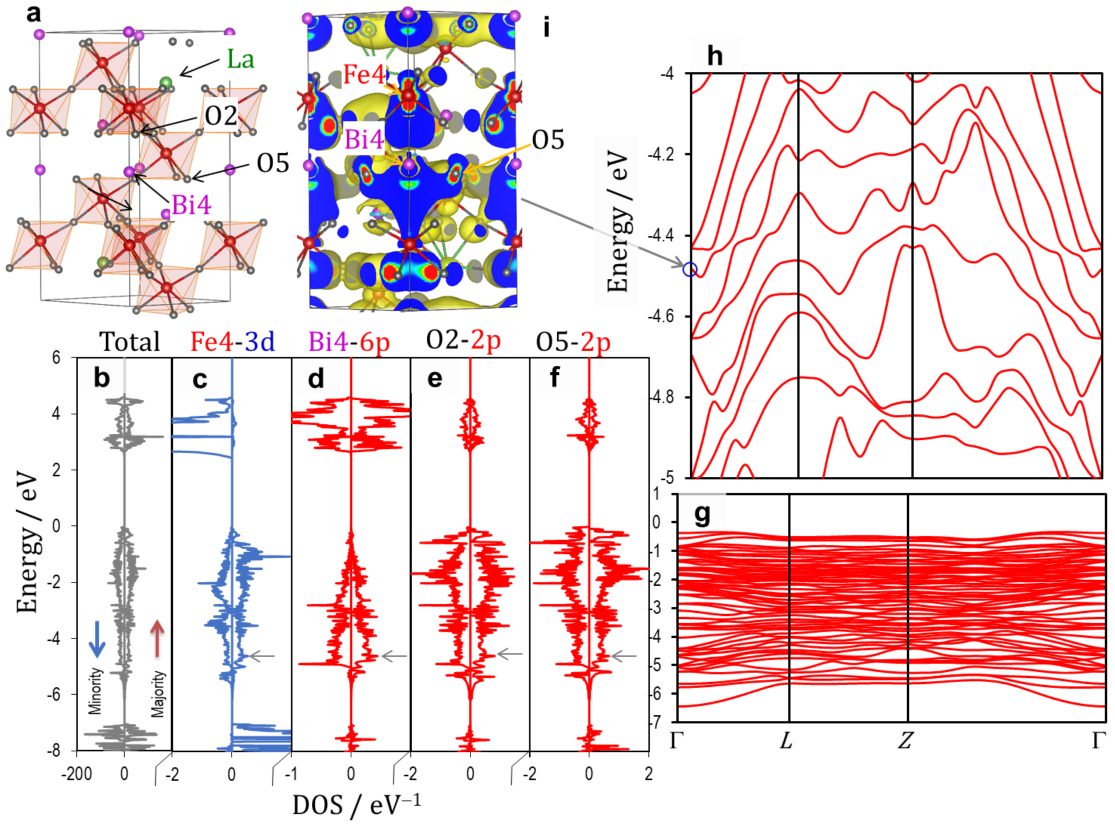

Figure S3. Crystal structures (**a**), electronic density of states (DOS) (**b**–**f**) and band structures (of the majority spin band) in the valence band (**g**, **h**) of the rhombohedral cell with $x = 2/6$. The wavefunction of the band shown in blue circle in **h** is displayed in **i**. The up (red) and down (blue) arrows in **b** denote the majority and minority spin bands, respectively, The horizontal arrows (gray) in **c**–**f** correspond to the energy level of the blue circle in **h**.

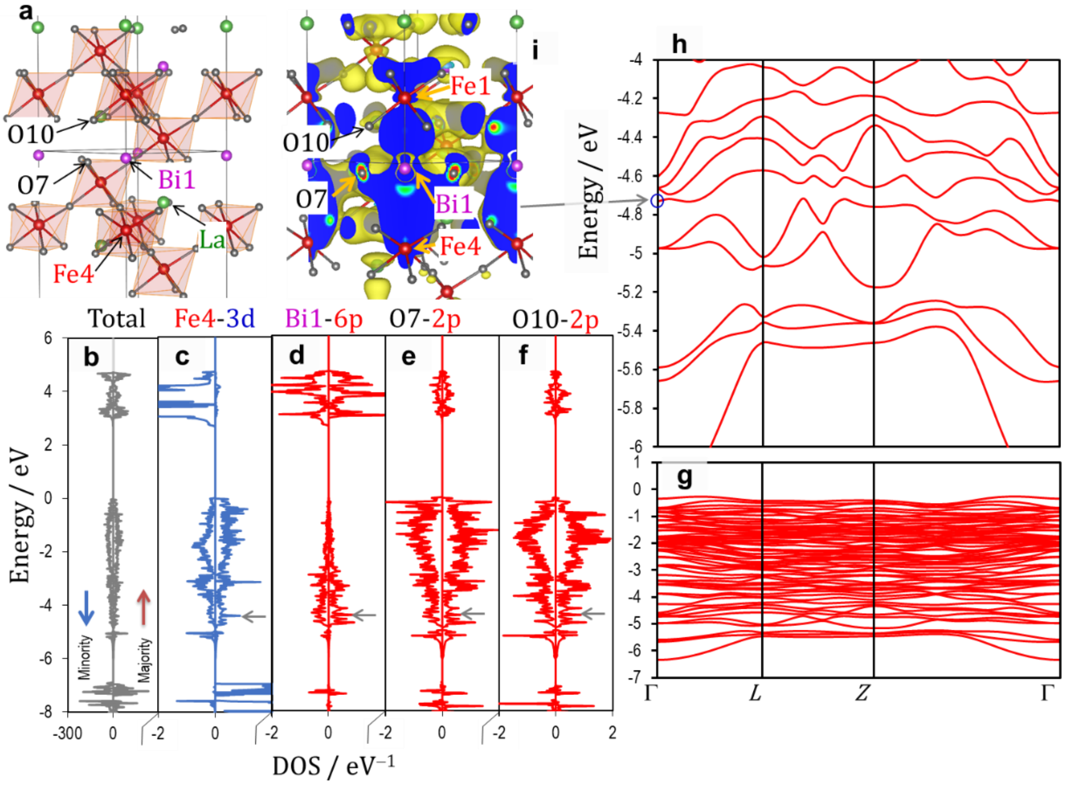

Figure S4. Crystal structures (**a**), electronic density of states (DOS) (**b**–**f**) and band structures (of the majority spin band) in the valence band (**g**, **h**) of the rhombohedral cell with $x = 4/6$. The wavefunction of the band shown in blue circle in **h** is displayed in **i**. The up (red) and down (blue) arrows in **b** denote the majority and minority spin bands, respectively, The horizontal arrows (gray) in **c**–**f** correspond to the energy level of the blue circle in **h**.

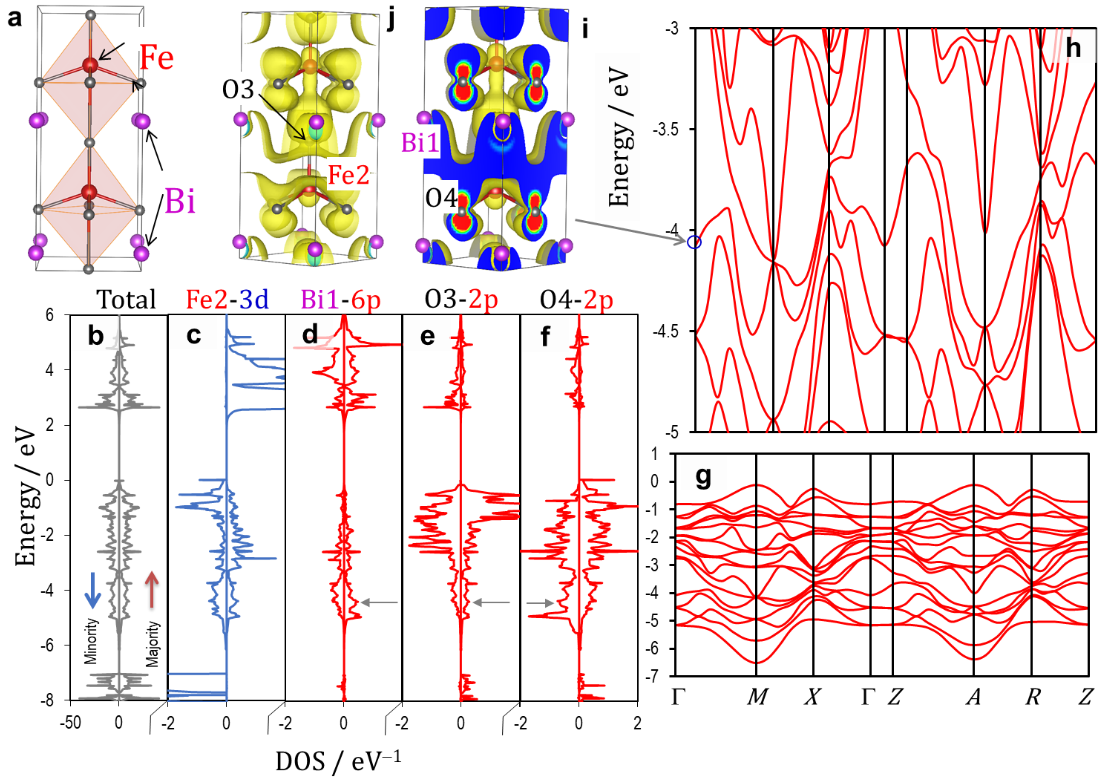

Figure S5. Crystal structures (**a**), electronic density of states (DOS) (**b**–**f**) and band structures (of the majority spin band) in the valence band (**g**, **h**) of the tetragonal $BiFeO_3$ cell with $x = 0$. The wavefunction of the band shown in blue circle in **h** is displayed in **j** and **i**. The up (red) and down (blue) arrows in **b** denote the majority and minority spin bands, respectively, The horizontal arrows (gray) in **c**–**f** correspond to the energy level of the blue circle in **h**.

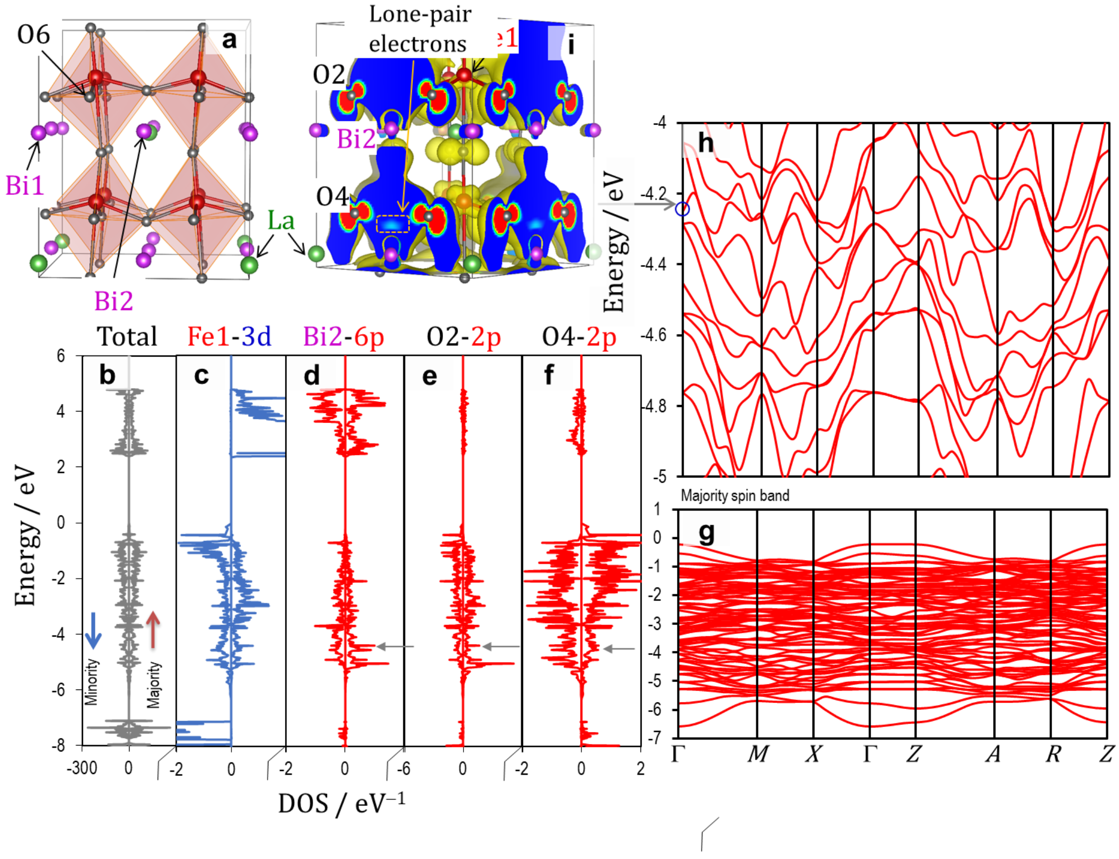

Figure S6. Crystal structures (**a**), electronic density of states (DOS) (**b**–**f**) and band structures (of the majority spin band) in the valence band (**g**, **h**) of the tetragonal cell with $x = 2/8$. The wavefunction of the band shown in blue circle in **h** is displayed in **i**. The up (red) and down (blue) arrows in **b** denote the majority and minority spin bands, respectively, The horizontal arrows (gray) in **c**–**f** correspond to the energy level of the blue circle in **h**.

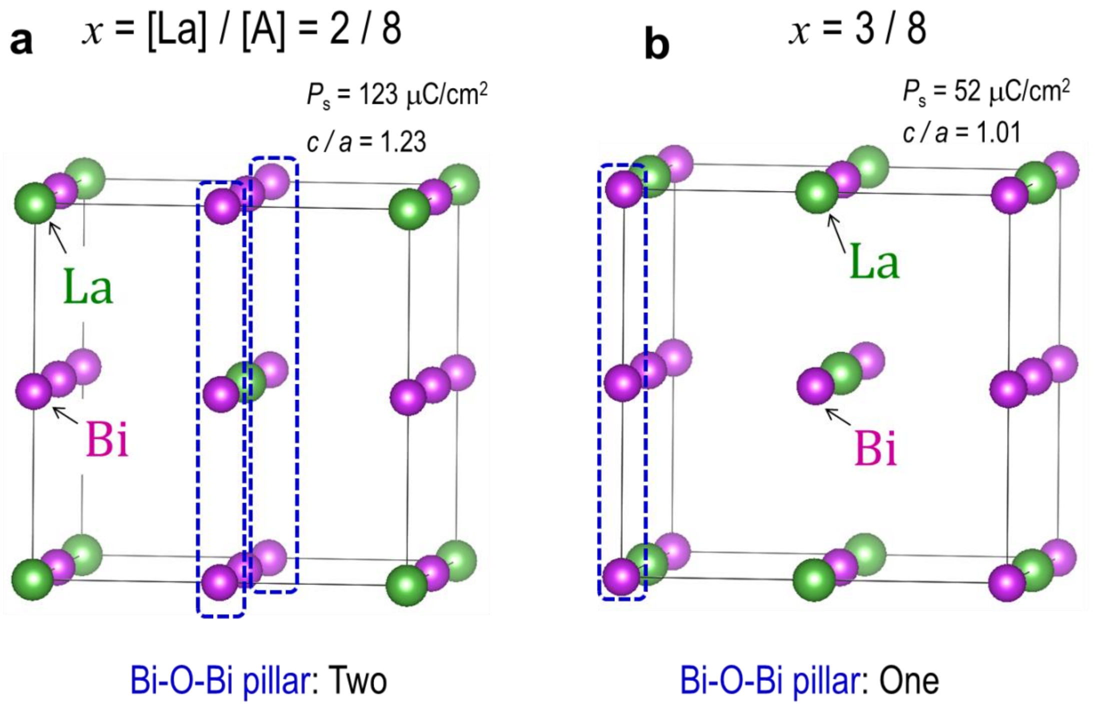

Figure S7. Bi and La arrangement along with Bi pillars along the polar *c* axis of the tetragonal cells with **a** $x = 2/8$ and **b** 3/8. In the tetragonal cell with $x = 2/8$, two Bi pillars remains and thereby a large P_s of 123 μC cm^{-2} associated with a high tetragonality (c/a) of 1.23 appear. An increase in La content (x) to 3/8 leads to a decrease in the Bi pillar to one, resulting in a marked decrease in P_s to 52 μC cm^{-2}. Its c/a is markedly reduced to 1.01 at $x = 3/8$.

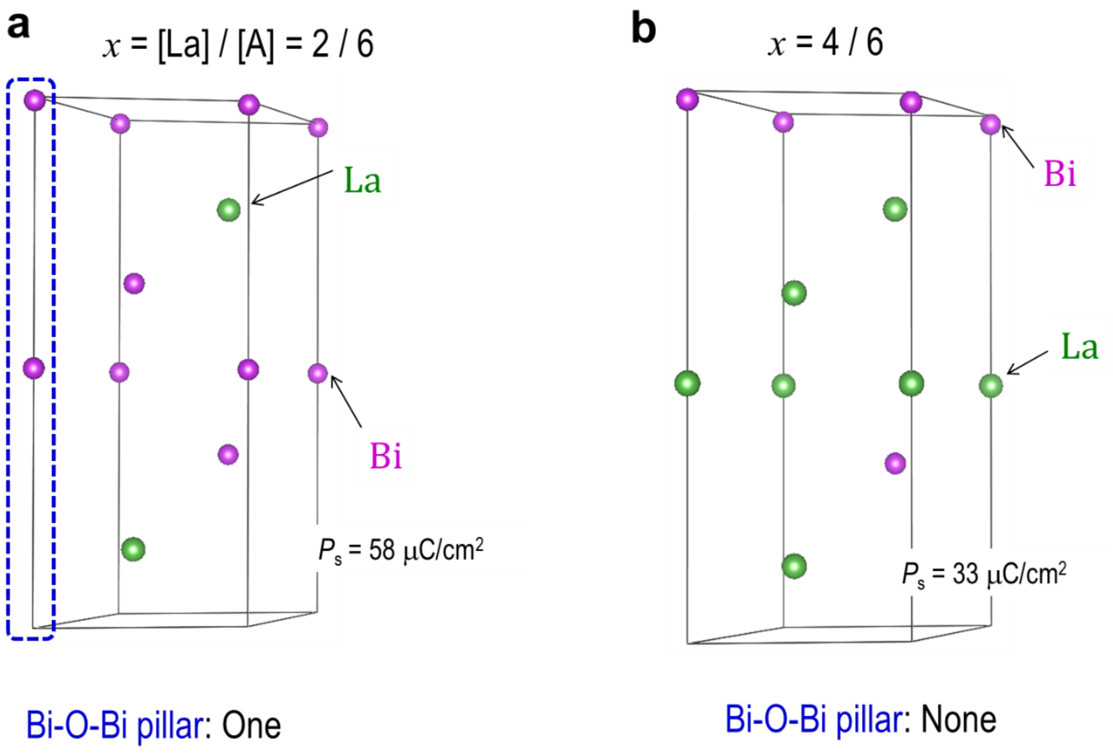

Figure S8. Bi and La arrangement along with Bi pillars along the polar *c* axis of the rhombohedral cells at **a** $x = 2/6$ and **b** 4/6. In the rhombohedral cell with $x = 2/6$, one Bi pillar is present and a relatively large P_s of 58 μC cm^{-2} appears. An increase in the La content (x) to 4/6 leads to a decrease in the Bi pillar to zero. The P_s monotonically decreases with increasing x: the rhombohedral cell $x = 4/6$ has a relatively large P_s of 33 μC cm^{-2} in spite of the absence of the Bi pillar.

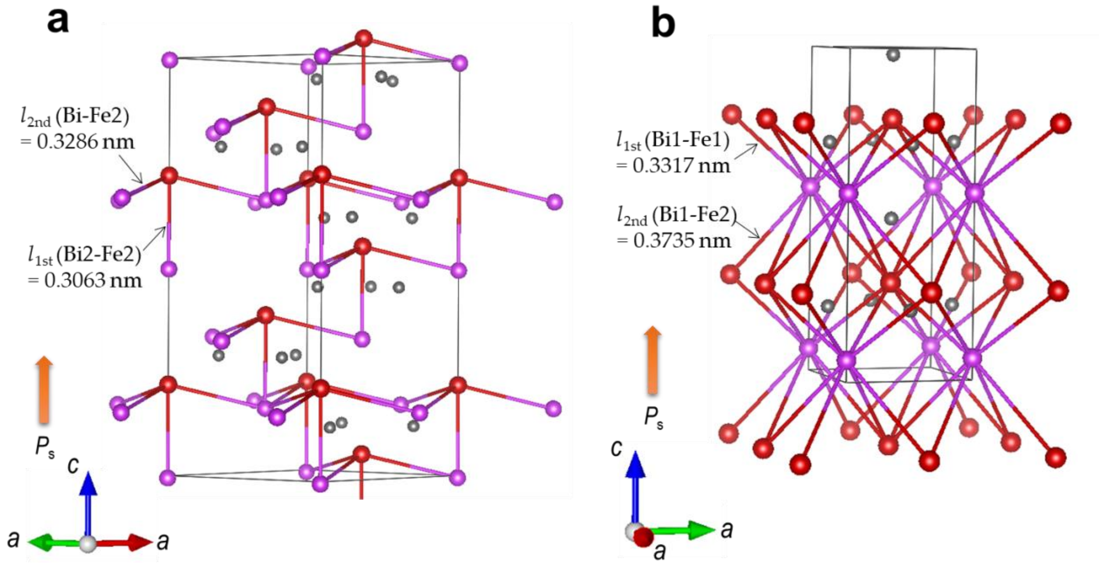

Figure S9. Bi-Fe bond lengths of $BiFeO_3$ in **a** rhombohedral and **b** tetragonal symmetries. Due to the distinct structural distortions, the 1st shortest Bi-Fe length (l_{1st}) and the 2nd shortest one (l_{1st}) are formed in the different directions with respect to their P_s vectors.

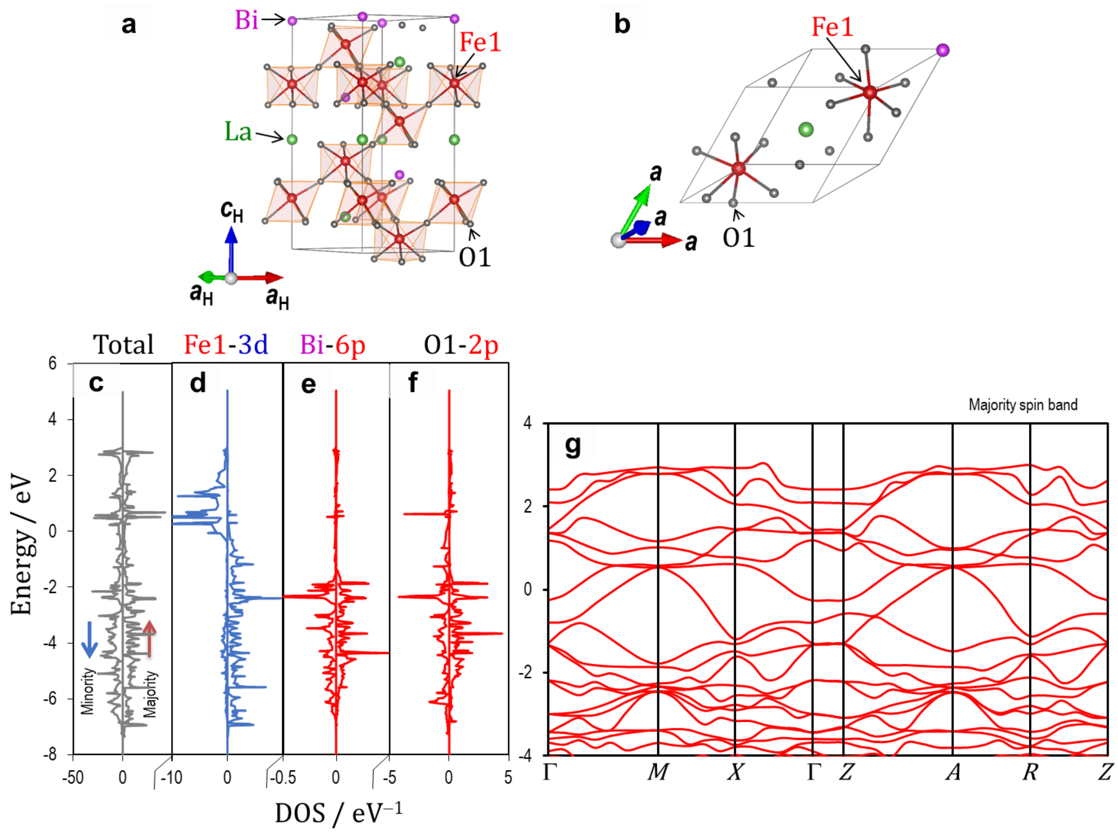

Figure S10. Crystal structures in the (**a**) hexagonal and (**b**) primitive settings, electronic density of states (DOS) (**c**–**f**) and band structure (of the majority spin band) in the valence band (**g**) of the rhombohedral (x = 1/2) with an ordered Bi-La arrangement along the polar ***c*** axis. The up (red) and down (blue) arrows in **c** denote the majority and minority spin bands, respectively. Note that the Fermi level is present inside the Fe-3*d* derived band and thereby this cell displays a metallic behavior. This is inconsistent with the experimental fact that these solid solutions exhibit an insulating nature. These results clearly show that not an ordered arrangement but a rock-salt one of Bi and La is preferred for our DFT calculations.
