# Supplementary material for: Origin of Ferroelectricity in BiFeO3-Based Solid Solutions
Source: Nanomaterials (Basel). 2022 Nov 24;12(23):4163. doi: 10.3390/nano12234163 (PMC9740055; doi:10.3390/nano12234163)
Supplement: Supplementary file 1 [file nanomaterials-12-04163-s001.zip › Supplementary_Tables.pdf]

Table S1. Empirical formula, space group, numbers of Bi and La, and bond lengths of Bi-Fe in the unit cells of the rhombohedral cells.

Empirical Formula	Z	Space Group [1)]	Number of Bi	Number of La	La content (x)	Bond Length of Bi-Fe / nm	
						1st Shortest	2nd Shortest
$BiFeO_3$	6	$R3$	6	0	0	0.3063	0.3286
$(Bi_5La)Fe_6O_{18}$	1	$P3$	5	1	1/6	0.3058	0.3286
						0.3102	0.3282
$(Bi_2La)Fe_3O_9$	2	$P3$	4	2	2/6	0.3058	0.3286
						0.3101	0.3298
						0.3102	0.3282
						0.3113	0.3299
$(BiLa)Fe_2O_6$	3	$R3$	3	3	3/6	0.3089	0.3293
$(BiLa_2)Fe_3O_9$	2	$P3$	2	4	4/6	0.3113	0.3291
						0.3119	0.3311
$(BiLa_5)Fe_6O_{18}$	1	$P3$	1	5	5/6	0.3213	0.3338
$LaFeO_3$	6	$R3$	0	6	1	0.3315 [2)]	0.3327 [2)]

1) Taking account of the antiferromagnetic spin configuration.
2) La-Fe length.

Table S2. Empirical formula, space group, numbers of Bi and La, and bond lengths of Bi-Fe in the unit cells of the tetragonal cells.

Empirical Formula	Z	Space Group[1)]	Number of Bi	Number of La	La content (x)	Bond Length of Bi-Fe / nm	
						1st Shortest	2nd Shortest
$BiFeO_3$	2	*P*4*mm*	2	0	0	0.3317	0.3735
$(Bi_7La)Fe_8O_{24}$	1	*P*4*mm*	7	1	1/8	0.3321	0.3742
						0.3315	0.3730
						0.3293	0.3784
						0.3309	0.3729
						0.3318	0.3711
$(Bi_3La)Fe_4O_{12}$	2	*P*4*mm*	6	2	2/8	0.3323	0.3722
						0.3290	0.3764
						0.3290	0.3764
						0.3323	0.3722
$(Bi_5La_3)Fe_8O_{24}$	1	*P*4*mm*	5	3	3/8	0.3256	0.3477
						0.3157	0.3650
						0.3225	0.3546
						0.3238	0.3585
$(BiLa)Fe_2O_6$	2	*P*4*mm*	4	4	4/8	0.3178	0.3640
						0.3178	0.3640
$(Bi_3La_5)Fe_8O_{24}$	1	*P*4*mm*	3	5	5/8	0.3235	0.3573
						0.3189	0.3594
$(BiLa_7)Fe_8O_{24}$	1	*P*4*mm*	1	7	7/8	0.3233	0.3532
$LaFeO_3$	2	*P*4*mm*	0	2	1	0.3346 [2)]	0.3369 [2)]

[1)] Taking account of the antiferromagnetic spin configuration.
[2)] La-Fe length.

Table S3. Empirical formula, space group, numbers of Bi and La, and bond lengths of Bi-Fe in the unit cells of the orthorhombic cells.

Empirical Formula	Z	Space Group[1)]	Number of Bi	Number of La	La content (x)	Bond Length of Bi-Fe / nm	
						1st Shortest	2nd Shortest
$BiFeO_3$	4	$P2_1m$	4	0	0	0.3182	0.3298
						0.3182	0.3298
$(BiLa)Fe_2O_6$	2	$P2_1m$	2	2	1/2	0.3186	0.3325
$LaFeO_3$	4	$P2_1m$	0	4	1	0.3241[2)]	0.3345[2)]
						0.3241[2)]	0.3345[2)]

1) Taking account of the antiferromagnetic spin configuration.
2) La-Fe length.

Table S4. Structural parameters of the rhombohedral $BiFeO_3$ cell (space group *R*3) with an antiferromagnetic spin configuration.

Lattice Parameter						
a	0.5528	nm	α	90	deg.	Density / Mg m^{-3}
b	0.5528	nm	β	90	deg.	8.595
c	1.3701	nm	γ	120	deg.	

Element	Multiplicity	Wyckoff Letter	Fractional Coordinates *x*	*y*	*z*	Magnetic Moment / μ_B
Bi1	3	*a*	0.0000	0.0000	0.9949	0.001
Bi2	3	*a*	0.0000	0.0000	0.4949	−0.001
Fe1	3	*a*	0.0000	0.0000	0.2184	−4.184
Fe2	3	*a*	0.0000	0.0000	0.7184	4.184
O1	9	*b*	0.3171	0.0905	0.6209	−0.024
O2	9	*b*	0.4401	0.4239	0.4542	0.023

Table S5 Structural parameters of the rhombohedral $(Bi_5La)Fe_6O_{18}$ cell (space group *P*3) with an antiferromagnetic spin configuration.

Lattice Parameter						
a	0.5529	nm	*α*	90	deg.	Density / Mg m^{-3}
b	0.5528	nm	*β*	90	deg.	8.333
c	1.3509	nm	*γ*	120	deg.	

Element	Multiplicity	Wyckoff Letter	Fractional Coordinates *x*	*y*	*z*	Magnetic Moment / μ_B
Bi1	1	*a*	0.0000	0.0000	0.0071	0.0010
Bi2	1	*b*	0.3333	0.6667	0.6768	−0.0010
Bi3	1	*c*	0.6667	0.3333	0.3441	0.0020
Bi4	1	*a*	0.0000	0.0000	0.5118	0.0000
La1	1	*b*	0.3333	0.6667	0.1890	0.0050
La2	1	*c*	0.6667	0.3333	0.8586	0.0040
Fe1	1	*a*	0.0000	0.0000	0.7774	−4.1730
Fe2	1	*b*	0.3333	0.6667	0.4504	−4.1790
Fe3	1	*c*	0.6667	0.3333	0.1137	−4.1700
Fe4	1	*a*	0.0000	0.0000	0.2823	4.1780
Fe5	1	*b*	0.3333	0.6667	0.9507	4.1760
Fe6	1	*c*	0.6667	0.3333	0.6190	4.1830
O1	3	*d*	0.7532	0.6544	0.7089	−0.0183
O2	3	*d*	0.0936	0.3256	0.3737	−0.0107
O3	3	*d*	0.4299	0.9943	0.0396	−0.0203
O4	3	*d*	0.0937	0.7763	0.8775	0.0200
O5	3	*d*	0.4171	0.4314	0.5434	0.0010
O6	3	*d*	0.7576	0.1073	0.2115	0.0217

Table S6. Structural parameters of the rhombohedral $(Bi_2La)Fe_3O_9$ cell (space group *P*3) with an antiferromagnetic spin configuration.

Lattice Parameter							
a	0.552877	nm	α	90	deg.	Density / Mg m^{-3}	
b	0.552877	nm	β	90	deg.	8.065	
c	1.350915	nm	γ	120	deg.		

Element	Multiplicity	Wyckoff Letter	Fractional Coordinates *x*	*y*	*z*	Magnetic Moment / μ_B
Bi1	1	*a*	0.0000	0.0000	0.0071	0.0010
Bi2	1	*b*	0.3333	0.6667	0.6768	−0.0010
Bi3	1	*c*	0.6667	0.3333	0.3441	0.0020
Bi4	1	*a*	0.0000	0.0000	0.5118	0.0000
La1	1	*b*	0.3333	0.6667	0.1890	0.0050
La2	1	*c*	0.6667	0.3333	0.8586	0.0040
Fe1	1	a	0.0000	0.0000	0.7774	−4.1730
Fe2	1	*b*	0.3333	0.6667	0.4504	−4.1790
Fe3	1	*c*	0.6667	0.3333	0.1137	−4.1700
Fe4	1	*a*	0.0000	0.0000	0.2823	4.1780
Fe5	1	*b*	0.3333	0.6667	0.9507	4.1760
Fe6	1	*c*	0.6667	0.3333	0.6190	4.1830
O1	3	*d*	0.7532	0.6544	0.7089	−0.0183
O2	3	*d*	0.0936	0.3256	0.3737	−0.0107
O3	3	*d*	0.4299	0.9943	0.0396	−0.0203
O4	3	*d*	0.0937	0.7763	0.8775	0.0200
O5	3	*d*	0.4171	0.4314	0.5434	0.0010
O6	3	*d*	0.7576	0.1073	0.2115	0.0217

Table S7. Structural parameters of the rhombohedral (BiLa)Fe_2O_6 cell (space group *R*3) with an antiferromagnetic spin configurations.

Lattice Parameters								
a	0.5532	nm	α	90	deg.			Density / Mg m^{-3}
b	0.5532	nm	β	90	deg.			7.779
c	1.3422	nm	γ	120	deg.			
Element	Multiplicity	Wyckoff Letter	Fractional Coordinates *x*	*y*	*z*			Magnetic Moment / μ_B
Bi	3	*a*	0.0000	0.0000	0.9889			0.000
La	3	*a*	0.0000	0.0000	0.4721			0.003
Fe1	3	*a*	0.0000	0.0000	0.2191			−4.173
Fe2	3	*a*	0.0000	0.0000	0.7152			4.177
O1	9	*b*	0.9049	0.2348	0.6288			−0.013
O2	9	*b*	0.5750	0.0152	0.4566			0.011

Table S8. Structural parameters of the rhombohedral $(BiLa_2)Fe_3O_9$ cell (space group *P*3) with an antiferromagnetic spin configuration.

Lattice Parameter								
a	0.5531	nm	*α*	90	deg.	Density / Mg m^{-3}		
b	0.5531	nm	*β*	90	deg.	7.490		
c	1.3361	nm	*γ*	120	deg.			

Element	Multiplicity	Wyckoff Letter	Fractional Coordinates *x*	*y*	*z*	Magnetic Moment / μ_B
Bi1	1	*a*	0.0000	0.0000	0.0160	0.0010
Bi2	1	*b*	0.3333	0.6667	0.6800	0.0000
La1	1	*c*	0.6667	0.3333	0.3603	−0.0040
La2	1	*a*	0.0000	0.0000	0.5300	0.0020
La3	1	*b*	0.3333	0.6667	0.1936	0.0040
La4	1	*c*	0.6667	0.3333	0.8680	0.0020
Fe1	1	*a*	0.0000	0.0000	0.7826	−4.1770
Fe2	1	*b*	0.3333	0.6667	0.4470	−4.1650
Fe3	1	*c*	0.6667	0.3333	0.1183	−4.1730
Fe4	1	*a*	0.0000	0.0000	0.2818	4.1720
Fe5	1	*b*	0.3333	0.6667	0.9506	4.1740
Fe6	1	*c*	0.6667	0.3333	0.6204	4.1730
O1	3	*d*	0.7589	0.6661	0.7028	−0.0020
O2	3	*d*	0.0981	0.3283	0.3716	−0.0217
O3	3	*d*	0.4282	0.9992	0.0360	−0.0010
O4	3	*d*	0.0916	0.7715	0.8749	0.0063
O5	3	*d*	0.4317	0.4385	0.5391	0.0077
O6	3	*d*	0.7581	0.1020	0.2076	0.0090

Table S9. Structural parameters of the rhombohedral $(BiLa_5)Fe_6O_{18}$ cell (space group *P*3) with an antiferromagnetic spin configuration.

Lattice Parameter								
a	0.5532	nm	α		90	deg.		Density / Mg m^{-3}
b	0.5532	nm	β		90	deg.		7.199
c	1.3288	nm	γ		120	deg.		
Element	Multiplicity	Wyckoff Letter	Fractional Coordinates *x*	*y*	*z*			Magnetic Moment / μ_B
Bi	1	*b*	0.3333	0.6667	0.6918			0.0000
La1	1	*a*	0.0000	0.0000	0.0325			−0.0010
La2	1	*c*	0.6667	0.3333	0.3648			−0.0020
La3	1	*a*	0.0000	0.0000	0.5352			0.0010
La4	1	*b*	0.3333	0.6667	0.1979			0.0010
La5	1	*c*	0.6667	0.3333	0.8666			0.0010
Fe1	1	*a*	0.0000	0.0000	0.7855			−4.1740
Fe2	1	*b*	0.3333	0.6667	0.4500			−4.1700
Fe3	1	*c*	0.6667	0.3333	0.1174			−4.1670
Fe4	1	*a*	0.0000	0.0000	0.2849			4.1690
Fe5	1	*b*	0.3333	0.6667	0.9493			4.1710
Fe6	1	*c*	0.6667	0.3333	0.6188			4.1720
O1	3	*d*	0.7583	0.6672	0.7015			0.0040
O2	3	*d*	0.0989	0.3320	0.3694			−0.0060
O3	3	*d*	0.4330	0.9983	0.0357			−0.0057
O4	3	*d*	0.0941	0.7669	0.8709			0.0010
O5	3	*d*	0.4324	0.4344	0.5363			0.0037
O6	3	*d*	0.7658	0.1010	0.2029			0.0037

Table S10. Structural parameters of the rhombohedral $LaFeO_3$ cell (space group $R3$) with an antiferromagnetic spin configuration.

Lattice Parameter								
a	0.5534	nm	α	90	deg.			Density / Mg m^{-3}
b	0.5534	nm	β	90	deg.			6.865
c	1.3285	nm	γ	120	deg.			
Element	Multiplicity	Wyckoff Letter	Fractional Coordinates					Magnetic Moment
			x	*y*	*z*			/ μ_B
La1	3	*a*	0.0000	0.0000	0.9657			0.000
La2	3	*a*	0.0000	0.0000	0.4657			0.000
Fe1	3	*a*	0.0000	0.0000	0.2152			−4.168
Fe2	3	*a*	0.0000	0.0000	0.7152			4.168
O1	9	*b*	0.9007	0.2339	0.6317			0.000
O2	9	*b*	0.5673	0.0001	0.4650			0.000

Table S11. Structural parameters of the tetragonal $BiFeO_3$ cell (space group *P*4*mm*) with an antiferromagnetic spin configuration.

Lattice Parameter						
a	0.3724	nm	α	90	deg.	Density / Mg m^{-3}
b	0.3724	nm	β	90	deg.	8.027
c	0.9335	nm	γ	90	deg.	

Element	Multiplicity	Wyckoff Letter	Fractional Coordinates *x*	*y*	*z*	Magnetic Moment / μ_B
Bi1	1	*a*	0.0000	0.0000	0.5672	−0.014
Bi2	1	*a*	0.0000	0.0000	0.0672	0.014
Fe1	1	*b*	0.5000	0.5000	0.7833	−4.206
Fe2	1	*b*	0.5000	0.5000	0.2833	4.206
O1	1	*b*	0.5000	0.5000	0.9791	−0.145
O2	2	*c*	0.5000	0.0000	0.7134	−0.117
O3	1	*b*	0.5000	0.5000	0.4791	0.145
O4	2	*c*	0.5000	0.0000	0.2134	0.117

Table S12. Structural parameters of the tetragonal $(Bi_7La)Fe_8O_{24}$ cell (space group *P*4*mm*) with an antiferromagnetic spin configuration.

Lattice Parameter						Density / Mg m^{-3}
a	0.7483	nm	α	90	deg.	7.772
b	0.7483	nm	β	90	deg.	
c	0.9283	nm	γ	90	deg.	

Element	Multiplicity	Wyckoff Letter	Fractional Coordinates *x*	*y*	*z*	Magnetic Moment / μ_B
Bi1	1	*a*	0.0000	0.0000	0.4345	−0.0170
Bi2	1	*a*	0.0000	0.0000	0.9317	0.0070
Bi3	2	*c*	0.5000	0.0000	0.4276	−0.0155
Bi4	2	*c*	0.5000	0.0000	0.9332	0.0140
Bi5	1	*b*	0.5000	0.5000	0.9374	0.0170
La2	1	*b*	0.5000	0.5000	0.4456	0.0240
Fe1	4	*d*	0.2488	0.2488	0.2163	−4.2100
Fe2	4	*d*	0.2517	0.2517	0.7191	4.2123
O1	4	*d*	0.2360	0.2360	0.0196	−0.1430
O2	4	*e*	0.2430	0.0000	0.2951	−0.1080
O3	4	*d*	0.2718	0.2718	0.5233	0.1455
O4	4	*e*	0.2554	0.0000	0.7780	0.1203
O5	4	*f*	0.2381	0.5000	0.2733	−0.1355
O6	4	*f*	0.2549	0.5000	0.7948	0.1150

Table S13. Structural parameters of the tetragonal $(Bi_3La)Fe_4O_{12}$ cell (space group *P*4*mm*) with an antiferromagnetic spin configuration.

Lattice Parameter						
a	0.7516	nm	α	90	deg.	Density / Mg m^{-3}
b	0.7516	nm	β	90	deg.	7.539
c	0.9212	nm	γ	90	deg.	

Element	Multiplicity	Wyckoff Letter	Fractional Coordinates *x*	*y*	*z*	Magnetic Moment / μ_B
Bi1	1	*a*	0.0000	0.0000	0.4375	−0.0170
Bi2	2	*c*	0.5000	0.0000	0.4293	−0.0155
Bi3	2	*c*	0.5000	0.0000	0.9293	0.0155
Bi4	1	*b*	0.5000	0.5000	0.9375	0.0170
La1	1	*a*	0.0000	0.0000	0.9446	−0.0270
La2	1	*b*	0.5000	0.5000	0.4446	0.0270
Fe1	4	*d*	0.2485	0.2485	0.2186	−4.2127
Fe2	4	*d*	0.2515	0.2515	0.7186	4.2127
O1	4	*d*	0.2259	0.2259	0.0215	−0.1430
O2	4	*e*	0.2420	0.0000	0.2974	−0.1098
O3	4	*d*	0.2741	0.2741	0.5215	0.1430
O4	4	*e*	0.2634	0.0000	0.7710	0.1350
O5	4	*f*	0.2366	0.5000	0.2710	−0.1350
O6	4	*f*	0.2581	0.5000	0.7974	0.1098

Table S14. Structural parameters of the tetragonal $(Bi_5La_3)Fe_8O_{24}$ cell (space group *P*4*mm*) with an antiferromagnetic spin configuration.

Lattice Parameter						
a	0.7805	nm	α	90	deg.	Density / Mg m^{-3}
b	0.7805	nm	β	90	deg.	7.967
c	0.7844	nm	γ	90	deg.	

Element	Multiplicity	Wyckoff Letter	Fractional Coordinates *x*	*y*	*z*	Magnetic Moment / μ_B
Bi1	1	*a*	0.0000	0.0000	0.4738	-0.0140
Bi2	1	*a*	0.0000	0.0000	0.9335	−0.0110
Bi3	2	*c*	0.5000	0.0000	0.4573	−0.0065
Bi4	1	*b*	0.5000	0.5000	0.9599	0.0170
La1	2	*c*	0.5000	0.0000	0.9755	0.0000
La2	1	*b*	0.5000	0.5000	0.4584	0.0230
Fe1	4	*d*	0.2459	0.2459	0.2447	−4.2432
Fe2	4	*d*	0.2512	0.2512	0.7412	4.2478
O1	4	*d*	0.2121	0.2121	0.0047	−0.0460
O2	4	*e*	0.2418	0.0000	0.3078	−0.1187
O3	4	*d*	0.2869	0.2869	0.5016	0.0340
O4	4	*e*	0.2651	0.0000	0.7305	0.1378
O5	4	*f*	0.2186	0.5000	0.2307	−0.1215
O6	4	*f*	0.2610	0.5000	0.8033	0.1135

Table S15. Structural parameters of the tetragonal (BiLa)Fe_2O_6 cell (space group *P*4*mm*) with an antiferromagnetic spin configuration.

Lattice Parameters						
a	0.5476	nm	α	90	deg.	Density / Mg m^{-3}
b	0.5476	nm	β	90	deg.	7.669
c	0.8024	nm	γ	90	deg.	

Element	Multiplicity	Wyckoff Letter	Fractional Coordinates *x*	*y*	*z*	Magnetic Moment / μ_B
Bi1	1	*a*	0.0000	0.0000	0.0580	0.013
Bi2	1	*b*	0.5000	0.5000	0.5580	−0.013
La1	1	*a*	0.0000	0.0000	0.5270	0.001
La2	1	*b*	0.5000	0.5000	0.0270	−0.001
Fe1	2	*c*	0.5000	0.0000	0.7590	−4.245
Fe2	2	*c*	0.5000	0.0000	0.2590	4.245
O1	2	*c*	0.5000	0.0000	0.9958	−0.050
O2	4	*d*	0.7427	0.7427	0.7296	−0.121
O3	2	*c*	0.5000	0.0000	0.4958	0.050
O4	4	*d*	0.7573	0.7573	0.2296	0.121

Table S16. Structural parameters of the tetragonal $(Bi_3La_5)Fe_8O_{24}$ cell (space group *P*4*mm*) with an antiferromagnetic spin configuration.

Lattice Parameter								
a	0.7820	nm	α		90	deg.		Density / Mg m^{-3}
b	0.7820	nm	β		90	deg.		7.492
c	0.7800	nm	γ		90	deg.		
Element	Multiplicity	Wyckoff Letter	Fractional Coordinates *x*	*y*	*z*			Magnetic Moment / μ_B
Bi1	1	*a*	0.0000	0.0000	0.9609			0.0170
Bi2	2	*c*	0.5000	0.0000	0.4493			−0.0085
La1	1	*a*	0.0000	0.0000	0.4638			0.0180
La2	2	*c*	0.5000	0.0000	0.9785			−0.0010
La3	1	*b*	0.5000	0.5000	0.5020			−0.0040
La4	1	*b*	0.5000	0.5000	0.9670			−0.0160
Fe1	4	*d*	0.2531	0.2531	0.2457			−4.2450
Fe2	4	*d*	0.2491	0.2491	0.7437			4.2475
O1	4	*d*	0.2835	0.2835	0.0029			-0.0320
O2	4	*e*	0.2754	0.0000	0.2313			−0.1190
O3	4	*d*	0.2161	0.2161	0.5013			0.0260
O4	4	*e*	0.2409	0.0000	0.8012			0.1115
O5	4	*f*	0.2534	0.5000	0.2966			−0.1283
O6	4	*f*	0.2481	0.5000	0.7273			0.1410

Table S17. Structural parameters of the tetragonal $(BiLa_7)Fe_8O_{24}$ cell (space group *P*4*mm*) with an antiferromagnetic spin configuration.

Lattice Parameter						
a	0.7818	nm	*α*	90	deg.	Density / Mg m^{-3}
b	0.7818	nm	*β*	90	deg.	7.062
c	0.7742	nm	*γ*	90	deg.	

Element	Multiplicity	Wyckoff Letter	Fractional Coordinates *x*	*y*	*z*	Magnetic Moment / μ_B
Bi	1	*a*	0.0000	0.0000	0.9661	0.012
La1	1	*a*	0.0000	0.0000	0.4491	0.018
La2	2	*c*	0.5000	0.0000	0.4883	−0.001
La3	2	*c*	0.5000	0.0000	0.9996	0.000
La4	1	*b*	0.5000	0.5000	0.4949	−0.003
La5	1	*b*	0.5000	0.5000	0.9819	−0.009
Fe1	4	*d*	0.2520	0.2520	0.2464	−4.244
Fe2	4	*d*	0.2490	0.2490	0.7473	4.247
O1	4	*d*	0.2861	0.2861	0.9997	−0.011
O2	4	*e*	0.2618	0.0000	0.2219	−0.129
O3	4	*d*	0.2149	0.2149	0.5010	0.016
O4	4	*e*	0.2479	0.0000	0.8000	0.120
O5	4	*f*	0.2496	0.5000	0.2914	−0.134
O6	4	*f*	0.2434	0.5000	0.7127	0.134

Table S18. Structural parameters of the tetragonal $LaFeO_3$ cell (space group *P*4*mm*) with an antiferromagnetic spin configuration.

Lattice Parameter						
a	0.3882	nm	α	90	deg.	Density / Mg m^{-3}
b	0.3882	nm	β	90	deg.	6.918
c	0.7734	nm	γ	90	deg.	

Element	Multiplicity	Wyckoff Letter	Fractional Coordinates *x*	*y*	*z*	Magnetic Moment / μ_B
La1	1	*a*	0.0000	0.0000	0.4959	0.000
La2	1	*a*	0.0000	0.0000	0.9959	0.000
Fe1	1	*b*	0.5000	0.5000	0.2485	−4.248
Fe2	1	*b*	0.5000	0.5000	0.7485	4.248
O1	1	*b*	0.5000	0.5000	0.9992	−0.002
O2	2	*c*	0.5000	0.0000	0.2500	−0.129
O3	1	*b*	0.5000	0.5000	0.4992	0.002
O4	2	*c*	0.5000	0.0000	0.7500	0.129

Table S19. Structural parameters of the orthorhombic $BiFeO_3$ cell (space group $P2_1/m$) with an antiferromagnetic spin configuration.

Lattice Parameter						
a	0.5598	nm	α	90	deg.	Density / Mg m^{-3}
b	0.7765	nm	β [1]	90	deg.	8.836
c	0.5410	nm	γ	90	deg.	

Element	Multiplicity	Wyckoff Letter	Fractional Coordinates *x*	*y*	*z*	Magnetic Moment / μ_B
Bi1	2	*e*	0.5497	0.2500	0.4901	0.001
Bi2	2	*e*	0.0495	0.2500	0.0098	−0.001
Fe1	2	*c*	0.0000	0.0000	0.5000	−4.213
Fe2	2	*b*	0.5000	0.0000	0.0000	4.213
O1	4	*f*	0.7963	0.4549	0.2003	−0.002
O2	4	*f*	0.2964	0.0448	0.2998	0.001
O3	2	*e*	0.9739	0.2500	0.5918	−0.111
O4	2	*e*	0.4734	0.2500	0.9082	0.111

[1] Geometrical optimization calculations were performed in monoclinic $P2_1/m$ for taking account of the antiferromagnetic spin configuration but the monoclinic angle β was set to 90 deg. and thereby the crystal system can be regarded as orthorhombic

Table S20. Structural parameters of the orthorhombic $(BiLa)Fe_2O_6$ cell (space group $P2_1/m$) with an antiferromagnetic spin configuration.

Lattice Parameters							
a	0.5558	nm	α	90	deg.	Density / Mg m^{-3}	
b	0.7806	nm	β [1]	90	deg.	7.807	
c	0.5448	nm	γ	90	deg.		

Element	Multiplicity	Wyckoff Letter	Fractional Coordinates x	y	z	Magnetic Moment / μ_B
Bi	2	*e*	0.0470	0.2500	0.0081	−0.002
La	2	*e*	0.5404	0.2500	0.4908	−0.001
Fe1	2	*c*	0.0000	0.0000	0.5000	−4.207
Fe2	2	*b*	0.5000	0.0000	0.0000	4.208
O1	4	*f*	0.7913	0.4549	0.2086	−0.004
O2	4	*f*	0.2929	0.0374	0.2941	0.003
O3	2	*e*	0.9857	0.2500	0.5877	−0.113
O4	2	*e*	0.4728	0.2500	0.9251	0.117

[1] Geometrical optimization calculations were performed in monoclinic $P2_1/m$ for taking account of the antiferromagnetic spin configuration but the monoclinic angle β was set to 90 deg. and thereby the crystal system can be regarded as orthorhombic

Table S21. Structural parameters of the orthorhombic $LaFeO_3$ cell (space group $P2_1/m$) with an antiferromagnetic spin configuration.

Lattice Parameter						
a	0.5514	nm	α	90	deg.	Density / Mg m^{-3}
b	0.7790	nm	β [1]	90	deg.	6.817
c	0.5507	nm	γ	90	deg.	

Element	Multiplicity	Wyckoff Letter	Fractional Coordinates *x*	*y*	*z*	Magnetic Moment / μ_B
La1	2	*e*	0.4699	0.2500	0.5070	0.002
La2	2	*e*	0.9697	0.2500	0.9929	−0.002
Fe1	2	*c*	0.0000	0.0000	0.5000	−4.202
Fe2	2	*b*	0.5000	0.0000	0.0000	4.202
O1	4	*f*	0.7810	0.9593	0.2196	−0.003
O2	4	*f*	0.2809	0.5415	0.2802	0.003
O3	2	*e*	0.0159	0.2500	0.4224	−0.120
O4	2	*e*	0.5144	0.2500	0.0776	0.120

[1] Geometrical optimization calculations were performed in monoclinic $P2_1/m$ for taking account of the antiferromagnetic spin configuration but the monoclinic angle β was set to 90 deg. and thereby the crystal system can be regarded as orthorhombic
